# Persistent Airway Hyperresponsiveness Following Recovery from Infection with Pneumonia Virus of Mice

**DOI:** 10.3390/v13050728

**Published:** 2021-04-22

**Authors:** Ajinkya R. Limkar, Caroline M. Percopo, Jamie L. Redes, Kirk M. Druey, Helene F. Rosenberg

**Affiliations:** 1 Inflammation Immunobiology Section, Laboratory of Allergic Diseases, National Institute of Allergy and Infectious Diseases, National Institutes of Health, Bethesda, MD 20892, USA; limka001@umn.edu (A.R.L.); percopoc@niaid.nih.gov (C.M.P.); 2 Lung and Vascular Inflammation Section, Laboratory of Allergic Diseases, National Institute of Allergy and Infectious Diseases, National Institutes of Health, Bethesda, MD 20892, USA; jamieredes@gmail.com (J.L.R.); kdruey@niaid.nih.gov (K.M.D.)

**Keywords:** respiratory virus, sublethal infection, inflammation, airway hyperresponsiveness

## Abstract

Respiratory virus infections can have long-term effects on lung function that persist even after the acute responses have resolved. Numerous studies have linked severe early childhood infection with respiratory syncytial virus (RSV) to the development of wheezing and asthma, although the underlying mechanisms connecting these observations remain unclear. Here, we examine airway hyperresponsiveness (AHR) that develops in wild-type mice after recovery from symptomatic but sublethal infection with the natural rodent pathogen, pneumonia virus of mice (PVM). We found that BALB/c mice respond to a limited inoculum of PVM with significant but reversible weight loss accompanied by virus replication, acute inflammation, and neutrophil recruitment to the airways. At day 21 post-inoculation, virus was no longer detected in the airways and the acute inflammatory response had largely resolved. However, and in contrast to most earlier studies using the PVM infection model, all mice survived the initial infection and all went on to develop serum anti-PVM IgG antibodies. Furthermore, using both invasive plethysmography and precision-cut lung slices, we found that these mice exhibited significant airway hyperresponsiveness at day 21 post-inoculation that persisted through day 45. Taken together, our findings extend an important and versatile respiratory virus infection model that can now be used to explore the role of virions and virion clearance as well as virus-induced inflammatory mediators and their signaling pathways in the development and persistence of post-viral AHR and lung dysfunction.

## 1. Introduction

While some respiratory virus infections present with comparatively mild symptoms, others can be severe and result in a substantial and long-term impact on lung function [1,2,3,4,5]. Recurrent wheezing and asthma are common complications observed in infants and children after recovery from severe respiratory syncytial virus (RSV) bronchiolitis [6,7,8,9,10,11,12]. For example, both Ruotsalainen et al. [9] and Stein et al. [10] found that many infants and young children diagnosed with severe RSV infections went on to develop symptoms of asthma that persisted into early or mid-to-late adolescence. Similarly, results from studies performed by Yoshihara et al. [11] and Mochizuki et al. [12] suggested that immunoprophylaxis against RSV resulted in reductions in the severity of acute illness as well as the incidence of subsequent recurrent wheezing. However, results from two recent meta-analyses revealed no conclusive evidence in support of a direct causal relationship between early-life RSV infection and subsequent respiratory dysfunction [13,14]. As such, the mechanisms underlying the observed clinical associations between RSV infection and respiratory dysfunction remain unclear.

Our group and others have modeled severe respiratory virus infection in wild-type mice in studies featuring the rodent-specific pathogen, pneumonia virus of mice (PVM; [15,16,17,18]). PVM and RSV are closely related viruses that are both members of the family *Pneumoviridae* and genus *Orthopneumovirus* [19]. However, unlike RSV, which replicates minimally and elicits few to no symptoms in most inbred strains of wild-type mice, PVM undergoes robust replication in mouse lung tissue and elicits severe symptoms associated with uncontrolled inflammation (i.e., the cytokine storm) [19,20,21,22]. Our findings and those of others have provided insight into the lethal impact of the PVM-associated cytokine storm as well as several global and pathway-specific blockade strategies that can be used to limit the sequelae of severe disease [21,23,24,25,26,27,28,29,30].

In this work, we aimed to examine outcomes in PVM-infected mice that experienced disease-associated symptoms but ultimately recovered from acute infection. Toward this end, we identified an inoculation strategy that resulted in significant but reversible weight loss and full seroconversion within 21 days of inoculation with no associated fatalities. We used this model to explore cytokine responses, leukocyte recruitment, and virus clearance, and to characterize the newly-identified dysfunctional airway responses that persisted in these mice well into the post-recovery period.

## 2. Materials and Methods

### 2.1. Mice

Wild-type BALB/c mice (6–10 weeks old) were purchased from Charles River Laboratories. Mice were maintained on-site in the 14BS vivarium at the National Institutes of Allergy and Infectious Diseases (NIAID) of the National Institutes of Health. All mouse studies were approved by the National Institute of Allergy and Infectious Diseases Animal Care and Use Committee and performed in accordance with Animal Study Protocol LAD-8E.

### 2.2. Virus Preparation and Inoculation Strategies

Pneumonia virus of mice (PVM) strain J3666 was prepared from clarified mouse-passaged stocks stored in liquid nitrogen. Mice under isoflurane anesthesia were inoculated intranasally with 50 µL PVM serially diluted in phosphate-buffered saline (PBS) with 0.1% bovine serum albumin (BSA) from 270 to 2.7 virus copies/mL as determined by a qPCR quantitation method as previously described [31]. Weights and survival were assessed daily in mice inoculated with 50 µL PVM at 270 or 27 copies/mL. Seroconversion in mice inoculated with sublethal doses of PVM was assessed at day 21 post-inoculation using the commercial Smart M12 kit (Biotech Trading Partners, Encinitas, CA, USA) as previously described [32]. Mice were sacrificed for evaluation at the time points indicated in each experiment. Bronchoalveolar lavage (BAL) was collected via two washes, each with 0.8 mL phosphate-buffered saline (PBS) with 0.1% bovine serum albumin (BSA). BAL fluid was clarified by centrifugation and stored at −80 °C until analysis; cells were collected and stored in freezing medium (90% fetal bovine serum (FBS) with 10% dimethyl sulfoxide) until evaluated flow cytometry as described below.

### 2.3. Profiling and ELISAs

Relative expression of proinflammatory and immunomodulatory mediators in BAL fluid of mice inoculated with a lethal vs. sublethal dose of PVM (day 7) and mice inoculated with a sublethal dose of PVM alone (day 7 vs. day 21) was evaluated using the Proteome Profiler Array, Panel A and the Mouse Angiogenesis Profiler Array (both from R&D Systems, Minneapolis, MN, USA) as previously described [33]. Each profiler membrane was probed with 500 µL BAL fluid (100 µL from each of 5 mice per group). Raw data were normalized to levels of each cytokine detected in BAL fluid samples from uninfected controls. Quantitative evaluation of proinflammatory cytokines in BAL fluid was carried out by ELISA (DuoSet, R&D Systems).

### 2.4. Histology

Lungs were perfused with 12 mL of PBS and inflated with 0.8 mL of cold 10% formaldehyde buffered-PBS before dissection. Dissected lungs were fixed overnight in 10 mL of 10% formaldehyde-buffered PBS solution at 4 °C. Formalin-fixed tissue was paraffin-embedded for slide preparation and subjected to de-paraffinization with xylene followed by ethanol and stained with hematoxylin and eosin by Histoserv, Inc. (Germantown, MD, USA). Images were generated using a Leica Slide Scanner.

### 2.5. Characterization of Leukocytes in BAL by Flow Cytometry

Cells were harvested from BAL by centrifugation and evaluated for myeloid subsets with Live/Dead Aqua (30 min at 4 °C) followed by anti-CD16/32 (BD Bioscience) and fluorochrome-conjugated anti-CD45 (clone 30-F11; eBioscience), anti-CD11c (clone N418; eBioscience), anti-MHC class II (clone M5/114.15.2; eBioscience), anti-Siglec F (clone E50-2440; BD Biosciences), and anti-Gr1 (clone RB6-8c5; BD Biosciences). Antibody staining was performed at 4 °C for 30 min and analyzed on the LSR II flow cytometer (Becton Dickinson, NJ, USA) with FlowJo software (Treestar, Woodburn, OR, USA). BAL leukocytes from uninfected (diluent control) mice were evaluated by visual inspection of modified-Giemsa-stained cytospin preparations.

### 2.6. Assessment of Bronchoconstriction with Precision Cut Lung Slices (PCLS)

Bronchoconstriction in PCLSs was performed as previously described [34,35]. Mice were euthanized at time points indicated by isoflurane inhalation. Cervical dislocation was performed carefully to avoid disruption of the trachea. The tracheae were exposed and transtracheal cuts were made using fine tip scissors. A blunt-ended 20-gauge needle was inserted into the cut and secured with silk suture. The lungs were inflated with 0.8 mL SeaPlaque low melting temperature agarose at 3% (weight/volume) in PBS. Mice were then covered in ice and placed in 4 °C for 1 h to allow the agarose to solidify. Following the incubation, the left lobe of the lung was dissected out and placed within the plunger of the Tissue Embedding Unit (Alabama Research and Development) together with additional 3% low melting agarose. The embedded tissues were allowed to solidify for 30 min at 4 °C, and they were then placed into a Krumdieck Tissue Slicer MD4000 (Alabama Research and Development) for preparation of 210 micron-thick slices. Slices with intact bronchi were washed three times on a shaking platform with PCLS wash medium (Ham’s F-12 medium with GlutaMax, 25 mM HEPES, 9 mM NaOH, and 1.5 mM CaCl_2_) for 30 min at 37 °C followed by incubation with PCLS wash medium overnight at 37 °C in a 5% CO_2_ incubator. Following the overnight incubation, slices were washed once more with PCLS Wash Medium for 30 min at 37 C on a shaking platform. Slices were individually weighted down in 24 well tissue culture plates using platinum wire-containing nylon threads (Harvard Apparatus, Holliston, MA, USA). Slices were then imaged using a Leica DMI4000 light microscope following exposure to either wash medium alone (control) or wash medium supplemented with increasing concentrations of carbachol (Cch; 10^−9^ to 10^−4^ M). Photographs (5× magnification) were taken at baseline and each dose of Cch. Bronchoconstriction was determined as the percentage of the luminal area remaining (over baseline) for individual bronchi that underwent constriction following stimulation with carbachol.

### 2.7. Measurements of Airway Resistance (FlexiVent)

Mice were anesthetized by intraperitoneal injection of a ketamine and xylazine cocktail (100 mg/kg and 10 mg/kg, respectively), followed by tracheal intubation with a 20-gauge cannula. The mice were mechanically ventilated at a rate of 150 breaths/min, a tidal volume of 10 mL/kg, and positive-end expiratory pressure of 3 cm H_2_O. Before the start of ventilation, mice were paralyzed by intraperitoneal injection of vecuronium bromide solution (0.1 mL of a 0.1 mg/mL solution). During data collection, PBS and increasing doses of methacholine (0, 3.125, 6.25, 12.5, and 25 mg/mL) were nebulized directly into the lungs of mice. Airway resistance was measured using the Scireq Flexivent system (Montreal, QC, Canada). Data presented are the means ± standard deviation (SD) of maximum resistance values (R_rs_) in units of cmH_2_O/mL/s. As the results obtained at day 21 largely paralleled those obtained by PCLS as described above, a day 45 trial was not performed.

### 2.8. Statistical Analyses

Statistical analyses were performed using algorithms in GraphPad Prism 8.0 as indicated in the respective Figure Legends; *p*-values < 0.05 are considered to be statistically significant.

## 3. Results

### 3.1. Symptomatic Sublethal PVM Infection in Wild-Type BALB/c Mice

**Figure 1:**
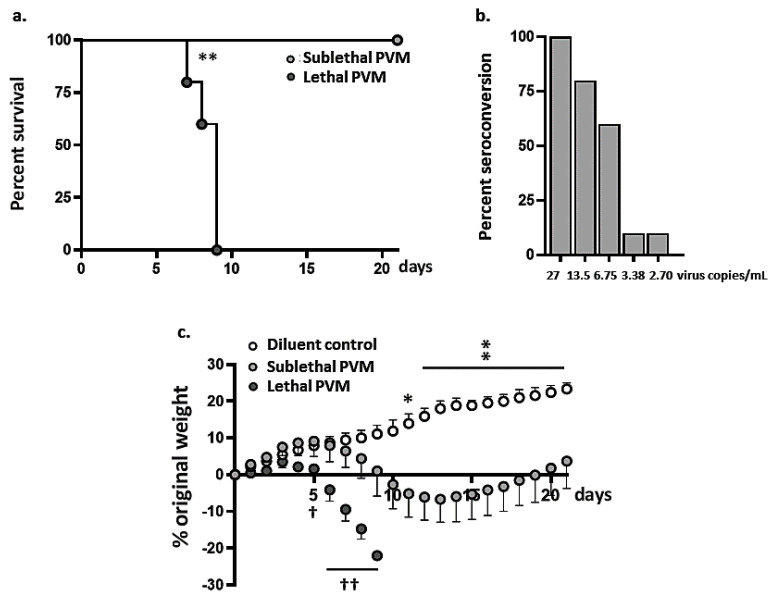
Sublethal PVM infection in wild-type BALB/c mice. (**a**) Survival of mice inoculated with a lethal (50 µL; 270 virus copies/mL) or sublethal (50 µL; 27 virus copies/mL) dose of PVM; *n* = 5 per group, ** *p* < 0.01, log-rank test. (**b**) Seroconversion at *t* = 21 days after inoculation with decreasing PVM titers as indicated; *n* = 5 per group. (**c**) Body weights of mice inoculated with lethal or sublethal doses of PVM as described in (**a**) or diluent alone; * *p* < 0.05, ** *p* < 0.01 for sublethal PVM versus diluent; ^†^ *p* < 0.05, ^††^ *p* < 0.01 for sublethal versus lethal PVM, 2-way ANOVA with Sidak’s post-hoc test.

To identify an inoculation strategy that would result in symptomatic but non-fatal PVM infection, we compared the outcomes resulting from inoculation using several reduced virus titers to the standard infection strategy. As shown in Figure 1a lethal infection results from inoculation of 50 µL at 270 virus copies/mL; the average survival time was 8.9 ± 0.9 days post-inoculation. By contrast, all mice inoculated with 50 µL at 27 virus copies/mL survived through day 21. Antibodies to PVM were detected at day 21 in all mice inoculated with this sublethal inoculum; by contrast, inoculation with virus titers that were further reduced (i.e., <27 copies/mL) did not result in full seroconversion (Figure 1b). Mice inoculated with the sublethal dose of PVM shown in Figure 1a experienced significant weight loss (to a maximum of 6.7 ± 2.8% of original body weight at day 13). As shown in Figure 1c, all mice inoculated with a sublethal dose of PVM ultimately recovered, although their weights remained significantly below those of matched uninfected mice throughout the course of the experiment (days 11–21). Mice inoculated with a lethal dose of PVM lost weight more rapidly and to a significantly greater degree than did mice inoculated with the sublethal dose.

**Figure 2:**
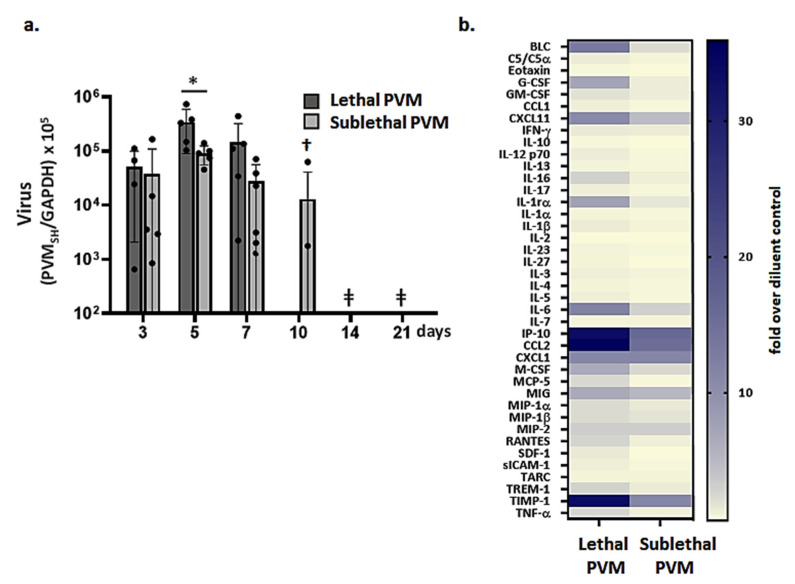
Virus replication and proinflammatory cytokines in sublethal PVM infection. (**a**) Virus detected in lung tissue by qPCR on days post-inoculation as indicated; * *p* < 0.05 sublethal vs. lethal PVM as in Figure 1a, 2-way ANOVA with Sidak’s post hoc test; ^†^ virus below detectable limits in *n* = 3 of 5 mice; ^ǂ^ virus below detectable limits in all 5 mice. (**b**) Cytokine profiling of BAL fluid; *n* = 5 mice per group. Data shown are fold increase over results from BAL fluid samples from uninfected controls.

Virus titers in lung tissue were evaluated on days 3–7 and 3–21 in response to inoculation with lethal or sublethal doses of PVM, respectively (Figure 2a). Although we detected more virus in lung tissue on day 5 after inoculation with a lethal compared to a sublethal dose (*p* < 0.05), we observed no dramatic differences in virus titers overall when comparing these two groups (days 3–7). This finding is consistent with our observations on PVM replication and clearance in mice treated with immunomodulatory *Lactobacillus plantarum*. Although administration of *L. plantarum* resulted in full protection against lethal PVM infection, this strategy had little to no impact on virus titers detected over time [28,29,30].

On day 10, virus was detected in lung tissue in only 40% of the mice inoculated with a sublethal dose of PVM (nb: none of the mice inoculated with a lethal dose of PVM survived to this time point, see Figure 1a); full virus clearance was observed on day 14. Substantially higher levels of proinflammatory cytokines were detected in bronchoalveolar lavage (BAL) fluid at day 7 in mice inoculated with a lethal versus a sublethal dose of PVM (Figure 2b). Among the most notable differences were IL-6, CCL2, and CXCL10 (IP-10), at 4.1-, 2.3- and 2.1-fold higher levels, respectively, than those detected in response to the sublethal PVM infection.

### 3.2. Resolution of Inflammation in Response to Sublethal PVM Infection

**Figure 3:**
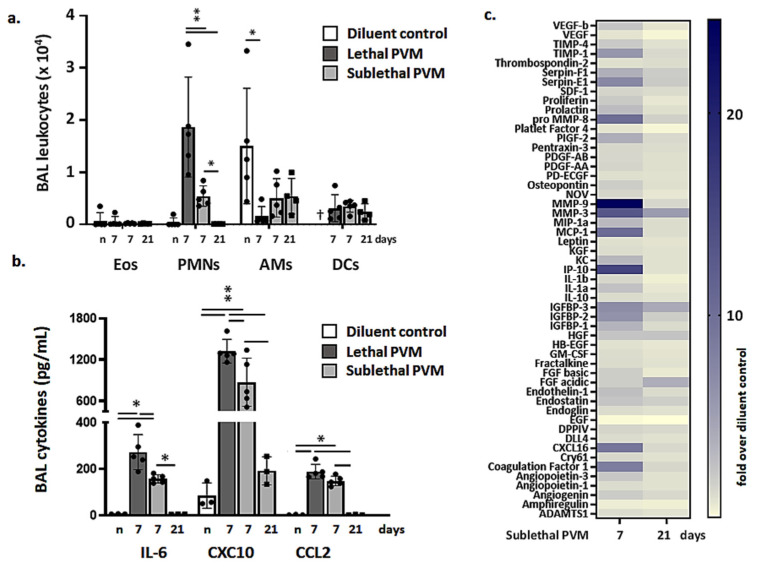
Resolution of inflammation in sublethal PVM infection. (**a**) Leukocytes, including eosinophils (Eos, CD11c^-^SiglecF^+^Gr1^lo^), neutrophils (PMNs, SiglecF^-^Gr1^hi^), alveolar macrophages (AMs, SiglecF^+^CD11c^+^), and dendritic cells (DCs, CD11c^+^MHCII^+^), detected in BAL fluid of uninfected mice (*n*; diluent control) and on days 7 and days 7 and 21 from mice inoculated with a lethal or sublethal dose of PVM, respectively; ^†^ no DCs were evaluated in mice were inoculated with diluent control (*n*); ** *p* < 0.01; unpaired *t*-tests. (**b**) Proinflammatory cytokines IL-6, CXCL10, and CCL2 in BAL fluid from uninfected mice (*n*; diluent control) and mice inoculated with a lethal or sublethal dose of PVM on days 7 or days 7 and 21, respectively; * *p* < 0.05, ** *p* < 0.01, unpaired *t*-tests. (**c**) Heat map documenting results of cytokine/mediator profiling performed on BAL fluid from mice inoculated with a sublethal dose of PVM; data shown (days 7 and 21) are fold-increases over results from uninfected controls, *n* = 5 mice per group.

Leukocytes recruited to the airways in response to lethal versus sublethal PVM infection were examined on days 7 and days 7 and 21, respectively. As shown in Figure 3a, significantly more neutrophils (SiglecF^-^Gr1^hi^ cells) were detected in BAL fluid from mice subjected to lethal PVM infection compared to those responding to a sublethal infection at the day 7-time point; few to no neutrophils remained by day 21. Similarly, significantly higher levels of critical cytokines and chemokines were detected at day 7 in BAL fluid from mice subjected to lethal PVM infection compared to those with sublethal infection (Figure 3b). All cytokines detected in BAL fluid in response to sublethal infection (day 7) returned to baseline levels by day 21. These findings were supported by mediator profiling as shown in the heatmap in Figure 3c.

**Figure 4:**
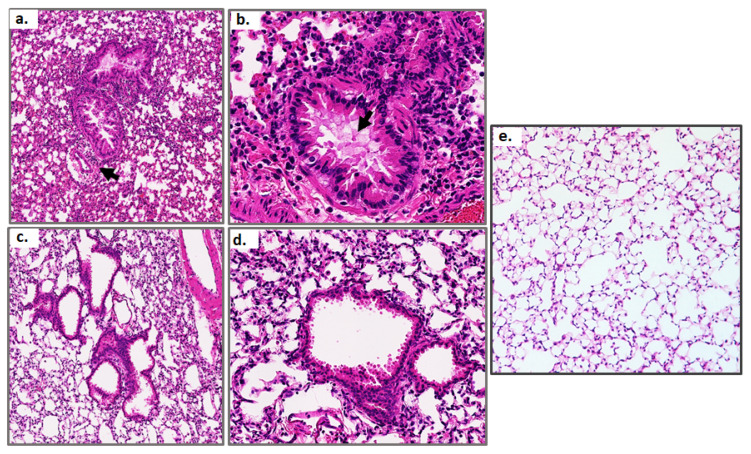
Lung histology at day 7 and day 21 of a sublethal PVM infection compared to uninfected control. Hematoxylin and eosin-stained lung tissue sections from mice inoculated with a sublethal dose of PVM and evaluated on day 7 ((**a**,**b**); original magnifications, 20× and 64×, respectively) and day 21 ((**c**,**d**); original magnifications, 20× and 64×, respectively); control lung tissue (**e**) at original magnification of 20×. Arrow in (**a**) indicates neutrophils surrounding a blood vessel adjacent to an airway; arrow in (**b**) points to pulmonary edema within the large airway. Each image is representative of findings from *n* = 5 mice.

Lung tissue sections from mice responding to a sublethal PVM infection were evaluated on days 7 and 21. Acute inflammation consisting primarily of neutrophils recruited to the lungs and airways was detected throughout the tissue on day 7. This was associated with mild-to-moderate edema of the larger airways (Figure 4a,b). Transmural migration of neutrophils from the vasculature was also detected in some sections. These responses were largely resolved by day 21 (Figure 4c,d). The tissue histology seen at day 21 post-inoculation can be compared to lung tissue from uninfected mice (diluent challenged; Figure 4e); the latter exhibited no inflammation and few to no neutrophils. Lung tissue histology from mice subjected to lethal infection was documented extensively in previous publications [15,16,32].

### 3.3. Airway Hyperresponsiveness after Recovery from Acute Sublethal PVM Infection

**Figure 5:**
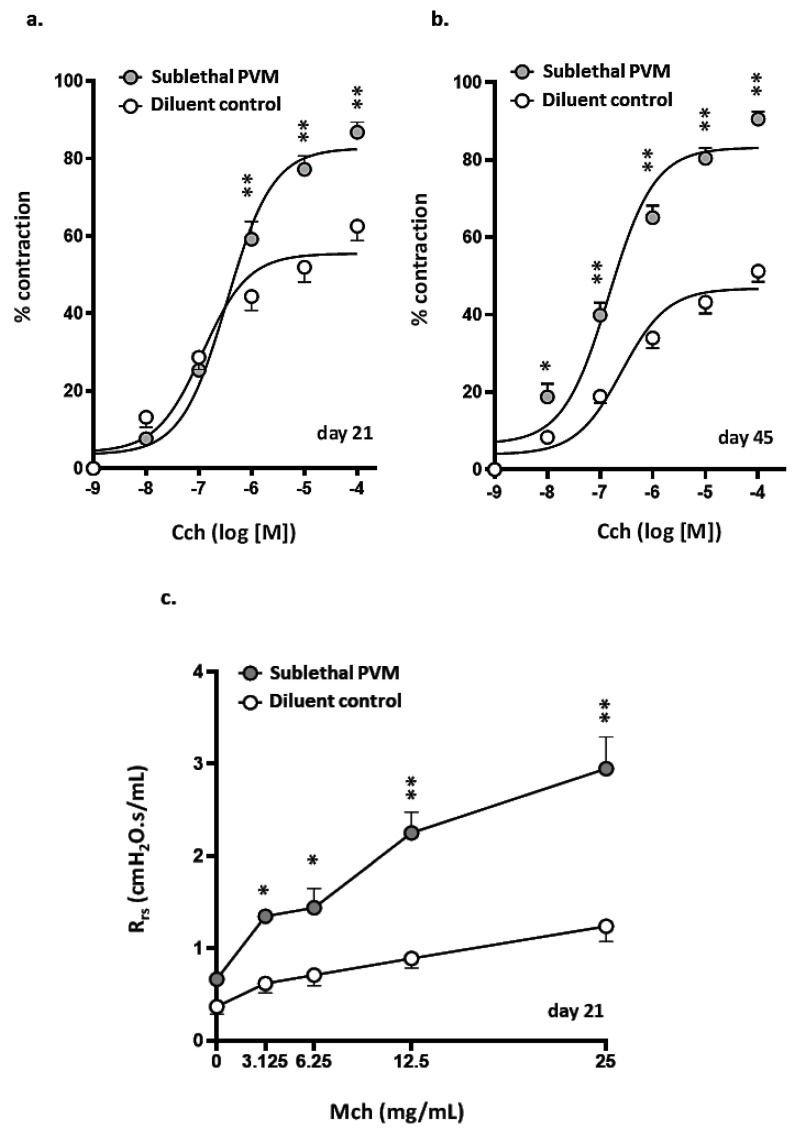
AHR after recovery from acute sublethal PVM infection. (**a**,**b**) Percent contraction observed in airways in response to increasing concentrations of carbachol (CCh) in precision-cut lung slices (PCLS) from uninfected mice and mice that recovered from an acute sublethal PVM infection; results on day 21 (**a**) and day 45 (**b**) are as shown; * *p* < 0.05, ** *p* < 0.01, 2-way ANOVA with Sidak’s post-hoc test; *n* = 5–8 mice per group. Sample data corresponding to (**b**) are shown in Supplemental Figure S1. (**c**) Total lung resistance (R_rs_) observed in response to administration of increasing concentrations of nebulized methacholine (MCh) to uninfected mice and mice that had recovered from an acute sublethal PVM infection (day 21); * *p* < 0.05, ** *p* < 0.01, 2-way ANOVA with Sidak’s post-hoc test, *n* = 4–5 mice per group.

Airway responses of mice that had recovered from a sublethal PVM infection were assessed on days 21 and 45 post-inoculation in PCLS challenged with increasing doses of carbachol (Cch). As shown in Figure 5a, target airways in PCLS prepared from mice that recovered from PVM infection (day 21) were significantly more responsive to Cch than were their counterparts from uninfected controls; increased potential for Cch-mediated contraction persisted through day 45 (Figure 5b and Supplemental Figure S1). Airway responses of mice that had recovered from sublethal PVM infection and those of uninfected controls were also assessed using invasive plethysmography. As shown in Figure 5c, total airway resistance (R_rs_) was significantly higher among mice that had recovered from a sublethal PVM infection compared to uninfected controls at all doses of methacholine (MCh).

## 4. Discussion

PVM (Family *Pneumoviridae*, genus *Orthopneumoviridae*) is a rodent-specific pathogen that is closely related to human RSV. Infection with PVM elicits responses in wild-type mice that largely phenocopy those of the most severe forms of RSV disease in human infants [15,16,17,18]. Studies carried out using the PVM infection model have underscored the critical contributions of the virus-induced cytokine storm and have explored ways that this response might be targeted effectively to prevent lethal disease. Among these findings, strategies directed at global [29,30] or pathway-specific [23,24,25,26,27,28] inhibition of virus-induced inflammation serve to reduce or, in some cases, completely prevent the lethal outcomes typically associated with PVM infection.

In this study, we developed a model of sublethal symptomatic PVM infection. Mice that received inoculations with reduced titer (i.e., a 10-fold reduction from a minimal lethal dose at constant volume) exhibited significant symptomatology, including reversible weight loss associated with virus replication, tissue inflammation, leukocyte recruitment, and seroconversion. We used this sublethal infection model to explore the resolution of inflammation and post-viral airway responses. BALB/c mice exhibited peak weight loss (~7% of original body weight) at day 13 after inoculation and ultimately returned to baseline weight by day 21. Recovery was accompanied by virus clearance and resolution of both biochemical and cellular inflammatory responses. Using both PCLS and invasive plethysmography, we found that mice that recovered from a sublethal PVM infection exhibited profound AHR that persisted up to 45 days after inoculation in response to challenge with bronchoconstrictors.

AHR is defined as intermittent and reversible airway narrowing and is manifested clinically as wheezing; shortness of breath; and in severe cases, hypoxia and death [36]. AHR results from both increased contraction of airway smooth muscle (ASM) in response to inflammatory mediators and airway remodeling resulting from inflammation [37]. In humans, AHR is diagnosed by the use of spirometry, which is a procedure that facilitates the estimation of airway resistance via measurements of the air volumes expelled during forced expiration at baseline and after challenge with bronchoconstrictors and/or bronchodilators [38]. As it is not possible to replicate this method in mice, investigators typically rely on plethysmographic techniques to measure lung resistance. In this manuscript, we present the results of studies carried out in vivo using invasive plethysmography, in which forced oscillations of air were administered to anesthetized animals via a cannulated trachea. Lung resistance in response to challenge with nebulized bronchoconstrictor agents was measured by a pressure transducer [39]. This technique is currently accepted as the most effective method available to determine lung resistance in mouse models of airway dysfunction [40]. We complemented these studies with PCLS experiments that permitted us to visualize airway contraction directly [34,35]. The results of these experiments demonstrated clearly that airways from mice that had recovered from sublethal PVM infection exhibited profound and persistent AHR.

AHR has also been reported in mouse models of influenza A infection [41] and in response to challenge with human RSV [42,43]. While RSV undergoes little to no replication and promotes minimal symptomatology in most inbred strains of mice, this pathogen has been used to model airways hyperreactivity and wheezing that develops in human infants after recovery from severe infection. Among these findings, van Schaik et al. [44] reported that BALB/c mice challenged with RSV exhibited higher respiratory rates and increased AHR to methacholine compared to uninfected controls. You et al. [45] reported that neonatal mice responded to a second challenge with the rA2-19F strain of RSV with airways hyperreactivity and pulmonary eosinophilia, which they attributed to an increase in viral load. Similarly, Estripeaut et al. [46] found that mice challenged with live RSV exhibited significant AHR up to 42 days after inoculation, correlating with viral persistence in lung tissue. However, in the case of PVM, we detected no viral persistence in lung tissue; instead, the mice exhibited significant AHR that persisted for several weeks after full virus clearance and resolution of the primary inflammatory response. An unbiased evaluation of the nature and extent of immunomodulatory dysregulation and tissue remodeling at or just before these later time points might provide insight into the mechanisms underlying AHR in this virus infection model.

We were also intrigued by the results of Rosas-Salazar et al. [47] who evaluated the nasopharyngeal microbiomes of 113 infants who recovered from an acute RSV infection. Among their findings, this group reported that the relative abundance of *Lactobacillus* species was consistently higher among infants who did not go on to develop post-viral wheezing. These findings are notable in light of previous work from our laboratory and those of others that documented that administration of immunobiotic *Lactobacillus* species directly to the respiratory tracts of mice served to inhibit virus-induced weight loss, inflammation, and in some cases, death, in response to acute respiratory virus infection [28,29,30,48,49]. Preliminary evidence from our lab suggests that administration of *Lactobacillus* species may also prevent the negative sequelae observed in response to sublethal infection (Limkar et al., manuscript in preparation).

We acknowledge several limitations of the work presented here. The study featured adult mice, while severe human RSV disease is largely an affliction of infants and young children [4,5]. Likewise, a more in-depth assessment of the impact of PVM infection on the responses of airway smooth muscle awaits further evaluation.

## 5. Conclusions

In summary, we identified a sublethal infection strategy that can be used to model the resolution of symptomatic PVM infection and to examine critical post-viral sequelae. We found that mice exhibit symptomatic infection with significant but reversible weight loss accompanied by virus replication and airway inflammation. Despite resolution of inflammation and virus clearance from the lungs and airways, mice that have recovered from a sublethal PVM infection exhibit persistent AHR when challenged with parasympathomimetic agents. Further evaluation of immune dysregulation after recovery from PVM infection may provide critical insight into the mechanisms underlying this response.

## Data Availability

All data are available from the corresponding author on request.

## Supplementary Materials

The following are available online at https://www.mdpi.com/article/10.3390/v13050728/s1, Figure S1: Airway contraction in precision-cut lung slices (PCLS).
