# Supplementary material for: Persistent Airway Hyperresponsiveness Following Recovery from Infection with Pneumonia Virus of Mice
Source: Viruses. 2021 Apr 22;13(5):728. doi: 10.3390/v13050728 (PMC8143513; doi:10.3390/v13050728)
Supplement: Supplementary file 1 [file viruses-13-00728-s001.zip › viruses-1184189-supplementary.pdf]

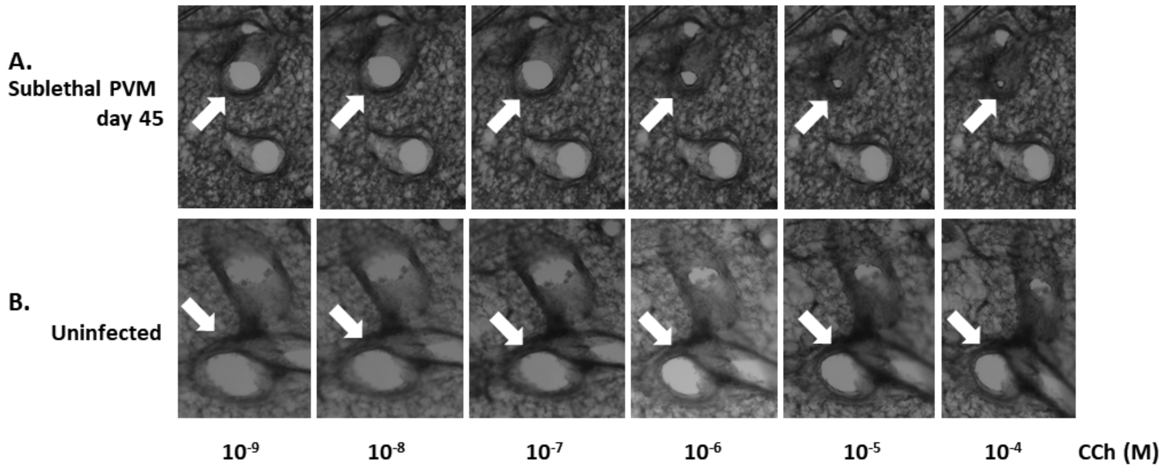

Supplemental Figure S1. Airway contraction in precision-cut lung slices (PCLS)

PCLS were prepared on day 45 from uninfected mice and mice inoculated on day 0 with a sublethal dose of PVM. As shown in (**A**), the target airway (indicated by white arrows) in a mouse that recovered from a sublethal PVM infection underwent profound contraction in response to increasing concentrations of carbachol (Cch), compared to (**B**) the limited responses of similar airways from uninfected mice. All images were prepared at 5x original magnification. Quantitative data are shown in Figure 5B.
